# Physiological basis and differentially expressed genes in the salt tolerance mechanism of *Thalassia hemprichii*

**DOI:** 10.3389/fpls.2022.975251

**Published:** 2022-11-28

**Authors:** Jie Shen, Zhongjie Wu, Lei Yin, Shiquan Chen, Zefu Cai, Xiaoxiao Geng, Daoru Wang

**Affiliations:** ^1^ Hainan Academy of Ocean and Fisheries Sciences, Haikou, China; ^2^ Key laboratory of Utilization and Conservation for Tropical Marine Bioresources, Hainan Tropical Ocean University, Ministry of Education, Sanya, China

**Keywords:** *Thalassia hemprichii*, salinity, differentially expressed genes, soluble sugar, soluble protein, malondialdehyde

## Abstract

Seagrass plays a vital role in the stability of marine ecology. The human development of marine resources has greatly affected the survival of seagrass. Seawater salinity is one of the important factors affecting its survival. Seagrass can survive in high saline environments for a long time and has evolved a variety of effective tolerance mechanisms. However, little is known about the molecular mechanisms underlying salinity tolerance by seagrass. *Thalassia hemprichii* is a seagrass species with a global distribution. It is also an ecologically important plant species in coastal waters. Nevertheless, the continuous environmental deterioration has gradually reduced the ecological niche of seagrasses. In this study, experiments were conducted to examine the effects of salinity changes on *T. hemprichii*. The result showed that the optimal salinity for *T. hemprichii* is 25 to 35 PSU. Although it can survive under high and low salinity, high mortality rates are common in such environments. Further analyses revealed that high salinity induces growth and developmental retardation in *T. hemprichii* and further causes yellowing. The parenchyma cells in *T. hemprichii* also collapse, the structure changes, soluble sugar accumulates rapidly, soluble proteins accumulate rapidly, the malondialdehyde (MDA) content reduces, and lipid peroxidation reduces in plant membranes. The molecular mechanisms of salt tolerance differ significantly between marine and terrestrial plants. We found 319 differentially expressed genes (DEGs). These genes regulate transport and metabolism, promoting environmental adaptation. The expression of these genes changed rapidly upon exposure of *T. hemprichii* to salinity stress for three hours. This is the first report on the physiological and biochemical changes and gene expression regulation of *T. hemprichii* under different salinity conditions. The findings of this study well deepen our understanding of *T. hemprichii* adaptations to changes in the shoal living environment.

## Introduction

Seagrasses are widely distributed along temperate and tropical coastlines of the world. They provide numerous ecosystem services: they can purify water quality, slow water flow, accumulate and stabilitate the sediment. In addition, they are habitats, breeding places, and food sources for marine life (Edgar et al., 1994; Orth et al., 2006). The global seagrass coverage is about 10% of the offshore area or 0.15% of the ocean area, yet the most productive and biodiverse marine ecosystem. To date, 74 seagrass species have been reported and widely distributed worldwide (Hemminga and Duarte, 2000; Huang et al., 2018). Global climate change and human activities severely affect seagrass beds (Waycott et al., 2009; Williams et al., 2016). Of these, salinity affects the health of seagrass beds and their long-term development, thus one of the critical factors in seagrass decline (Waycott et al., 2009). Rapidly expanding low-salinity aquaculture activity along shallow coastlines discharges low-salinity water with a high concentration of nutrients (Funge-Smith and Briggs, 1998; Heil, 2000; Cao et al., 2007). In addition, climate change has increased pulse-type heavy rainfall events (IPCC, 2007), dramatically increasing freshwater loads (Faxneld et al., 2010) and possibly affecting coastal seagrasses (Waycott et al., 2007). Such an extreme event has caused large-scale losses of seagrass habitats in Eastern Africa (Bandeira and Gell, 2003), Queensland of Australia (Campbell and McKenzie, 2004), and Venezuela (Chollett et al., 2007). On the other side, global warming has accelerated lagoon evaporations, and the silting at the mouth reduces seawater exchange, leading to high salinity in the lagoons. Seawater salinity affects the osmotic pressure of seagrass plant cells, disrupting their physiological and biochemical characteristics, thus, affecting seagrass survival (Munns, 2002). For example, high salinity stress decreases nonstructural sugars content, glutamate synthase activity and growth potential of *Posidonia oceanica* (Duarte et al., 2010). High salinity promotes the predominance of salt-tolerant algae, negatively affecting seagrass ecosystems (Huang et al., 2006).

To cope with salt stress, plants have evolved various strategies, such as changes in signaling pathways, accumulation of reactive oxygen species (ROS), activations in antioxidant systems, scavenging and repairing of redox balance, and induction or degradation of osmotic regulation, etc. Seagrasses originated on land, migrated to fresh or brackish water, and are the only higher plant living wholly submerged in the marine environment (Wissler et al., 2011). Salinity tolerance is a major evolutionary factor in physiological adaptation separating seagrasses from their freshwater relatives. The salt tolerance of *Zostera marina* is higher than that of terrestrial salt-tolerant plants. Meanwhile, its evolutionary position is higher than other lower salt-tolerant plants in the ocean. Salinity is a key ecological factor affecting the survival, growth, and distribution of seagrass. Due to their long-term survival in a high salinity environment, seagrasses have evolved various high-efficiency salt tolerance mechanisms to adapt to salinity fluctuations by modulating distinct physiological responses from intracellular ion concentration (e.g., organic osmotic regulators, etc.) to cell wall elasticity (Larkum et al., 2006; Touchette, 2007; Deng and Lü, 2018). Current research on the mechanism underlying salt tolerance in higher plants mainly focuses on terrestrial halophytes. However, the physiological and molecular mechanisms underlying the salt tolerance of seagrasses are poorly studied (Deng and Lü, 2018).

*T. hemprichii* is widely distributed in shallow coastal areas in the tropics and subtropics of the eastern Atlantic and Indo-Pacific, between 28°Sand 32°N (Den, 1970; Phillips and Meñez, 1988; Spalding et al., 2003). In the South of China Sea, *T. hemprichii* plays a vital role in balancing the ecological environment in the coastal waters. The presence of *T. hemprichii* affects marine ecology, biogeography, and genetic diversity (Lee et al., 2016; Nordlund et al., 2017; Jahnke et al., 2019). With the further deterioration of the marine ecological environment, extreme salinity fluctuations in coastal areas have led to restrained growth or even death of *T. hemprichii* (Sinclair et al., 2016; Wainwright et al., 2018; Shen et al., 2021). However, at present, the tolerance and mechanisms underlying the response of *T. hemprichii* to salinity stress have not been adequately studied. This study compared and analyzed the growth and development, measured physiological and biochemical indexes, and analyzed transcriptome information of *T. hemprichii* under different salinity conditions. The result of this study provides a research basis for the investigation of seagrass adaptability to salinity changes in a large environment.

## Materials and methods

### Plant materials and collection

**Figure 1:**
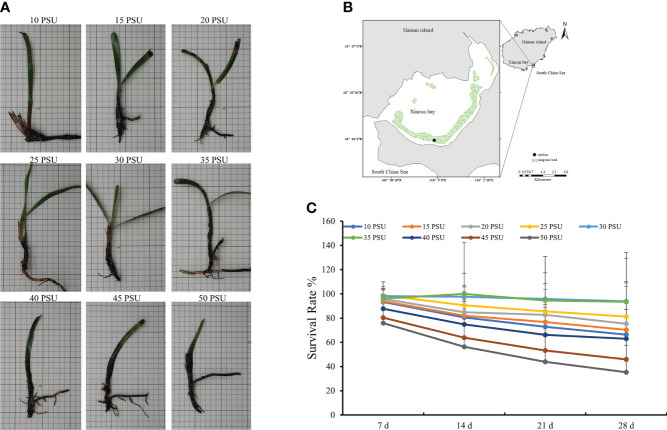
The growth state and survival rate of *T. hemprichii* under different salinity conditions. **(A)** Growth state; **(B)** Location of sampling site, Xincun Bay, South China Sea; **(C)** Survival Rate. The statistical significance was assessed using a one-way analysis of variance (ANOVA), *P* < 0.01.

*T. hemprichii* was collected from the Lingshui Xincun port seagrass special protected area, Hainan, China. Xincun Bay, a lagoon of about 21.97 km^2^, is located in the southeast of Lingshui Li Autonomous County, Hainan Province, and south to Li’an port. It is about 4 km long from north to south, with a port door from Xincunjiao (18°24′42′′N,109°57′58′′E) to Shitoucun Shazui (18°24′34′′N, 109°57′42′′E) (**Figure 1B**), an area exhibiting typically oceanic salinity around 28 to 32 PSU. Intact and robust plants were selected, rinsed with tap water to remove sediment and attachments on the plants, and taken to the laboratory.

The collected intact *T. hemprichii* plants were transplanted into a glass aquarium. The bottom of the glass aquarium was covered with aquarium soil and coral sand (1:1) and filled with 60 L seawater (salinity = 30 PSU). The culture temperature was 26 ± 1°C, and the light-dark cycle was 12L/12D. Salinity was increased or decreased using Instant Ocean salts and Deionization Water, and monitored twice daily using a Lab Salinometer (SYA2-2, National Ocean Technology Center, China). The medium was carefully renewed daily (Jiang et al., 2013).

### Plant treatments with different salinity conditions

Twenty-five plants were planted in each treatment, the culture temperature was 26 ± 1°C, and the light-dark cycle was 12L/12D. The *T. hemprichii* plants were harvested after 28 days of laboratory culture under 10, 15, 20, 25, 30, 35, 40, 45, and 50 PSU.

The salt tolerance threshold was determined every 7days. The salt tolerance threshold is the salt concentration of 50% survival rate (plants grow within this range; more than 50% of the plants can grow normally in this range. When salinity exceeds this threshold, the growth of more than 50% of the plants is restricted, reducing the yield). In this study, statistical analyses were performed based on the percentage of surviving leaves (dropped leaves and black leaves were considered dead) against the total number of leaves using fifteen plants per treatment. Proportional data were arcsine square root-transformed before analysis. All statistical analyses (student-t, p < 0.05) were performed using SPSS (v.21.0).

### Ultrastructure observation of plant

Three treatments with 15, 30, and 45 PSU were selected to observe the structure by optical microscope, scanning electron microscopy (SEM) and transmission electron microscopy (TEM). The underground stems and leaves of 0, 7, 14, 21, and 28 days were selected for sectioning to observe their microstructure and ultrastucture. Dehydrating, embedding, sectioning, and staining were performed by Wuhan Sevier Biotechnology Co., Ltd., Wuhan, China.

The specimens were observed under NIKON ECLIPSE E100 positive optical microscope (Nikon, Tokyo, Japan), HITACHI scanning electron microscope SU8100 (Hitachi, Tokyo, Japan), and HITACHI transmission electron microscope HT7800/HT7700 (Hitachi, Tokyo, Japan).

### Determination of plant resistance indicators

Three treatments with 15, 30, and 45 PSU salinity were selected to determine plant resistance characteristics. Samples were taken every 7 days after different salinity treatments. After sampling the leaves and wiping off the water, 0.1 g was weighed, quick-frozen in liquid nitrogen, and frozen at -80 °C to determine various physiological and biochemical indicators of *T. hemprichii*. The test period was 21 days. The experiments were replicated three times.

Physiological indicators, such as soluble protein, soluble sugar, malondialdehyde (MDA), catalase (CAT), peroxidase (POD), and superoxidase (SOD), were measured using the total protein assay kit (with standard: BCA method) (A045-3-2), plant soluble sugar content test kit (A145-1-1), malondialdehyde (MDA) assay kit (TBA method) (A003-1-2), catalase (CAT) assay kit (Visible light) (A007-1-1), peroxidase assay kit (A084-3-1), and total superoxide dismutase (T-SOD) assay kit (Hydroxylamine method) (A001-1-2) (Nanjing Jiancheng Biotechnology Engineering Institute, Nanjing, China), respectively.

### RNA-sequencing and analysis

Samples treated with 15, 30, and 45 PSU were selected for comparative transcriptome sequencing. After treatments, the samples were taken at 0, 3, 6, 12, 24, and 48 hours, respectively. Sequencing was conducted by Beijing Genomics institution (BGI-Shenzhen, China).

The mRNA library construction and transcriptome sequencing were performed by BGI, and the single end of the amplified flow cell was sequenced by the BGSEQ-500 Platform (BGI-Shenzhen, China). The raw reads were filtered using Trimmonatic software to get clean reads, then assembled by SOAPdenovo software. The transcripts were de-redundant to obtain unigenes. Next, the unigene sequences were compared with the protein databases, including the National Center for Biotechnology Information (NCBI) non-redundant (NR), Swiss-Prot, Kyoto Encyclopedia of Genes and Genomes (KEGG), and Clusters of Orthologous Groups of proteins (COG) by blastX (e-value < 0.00001). Sequence orientation of unigenes was determined using the protein sequences with the best comparison results. The sequence orientation of unigenes was determined in the priority of NR, Swiss-Prot, KEGG, and COG when results from different databases conflicted. Unigenes which could not be compared with any of the above four libraries were predicted using the ETScan software.

The expression levels of unigenes were calculated using the FPKM (Fragments Per Kilobase of transcript per Million) method, and differentially expressed genes (DEGs) between samples were determined by log2 (Fold change). Genes with log2 (Fold change) ≥ 1 and P-value < 0.05 were classified as up-regulated genes, while genes with log2 (Fold change) < 1 and P-value < 0.05 were classified as down-regulated genes.

### Quantitative real-time PCR

Quantitative real-time PCR assays for multiple genes were performed using the SYBR^®^ Premix Ex Taq™ II (Takara, Shiga, Japan). Two or three primer pairs were designed for all the amplification segments to ensure the qPCR quality. However, only one pair was used in the final test. The primer sequences are given in **Table S1**. Melting-curve analyses were performed for all the primers. The 18S rRNA was used as an internal reference gene to normalize the Ct values for each gene. Quantitative real-time PCR was performed by a Mastercycler^®^ ep realplex (Eppendorf, Hamburg, Germany). All qPCR assays were replicated three times to eliminate mechanical errors.

### Data analysis

Gene expression was analyzed using a one-way analysis of variance (ANOVA) with Tukey adjustments. The qPCR reactions and data were analyzed according to the methods of Livak and Schmittgen (2001) and Bustin et al. (2009). The data were analyzed with ANOVA to determine the treatment effects relative to the control.

## Results

### Growth and development of *T. hemprichii* under different salinity stresses

*T. hemprichii* had stunted growth, with yellow leaves under low salinity stress, and the plants died rapidly under high salinity stress (**Figure 1A**). The mortality rates of *T. hemprichii* under the three treatments with the salinity of 40, 45, and 50 PSU were over 40% after 28 days of treatments. With low salinity of 10, 15, and 20 PSU, plant death occurred after 21 days of treatments, and the mortality rates were above 30%. The mortality rates of *T. hemprichii* under salinity of 25 to 35 PSU did not exceed 10% (**Figure 1C**), and all plants grew normally with green leaves (**Supplementary Figure S1**).

### Ultrastructure of *T. hemprichii* under different salinity stresses

**Figure 2:**
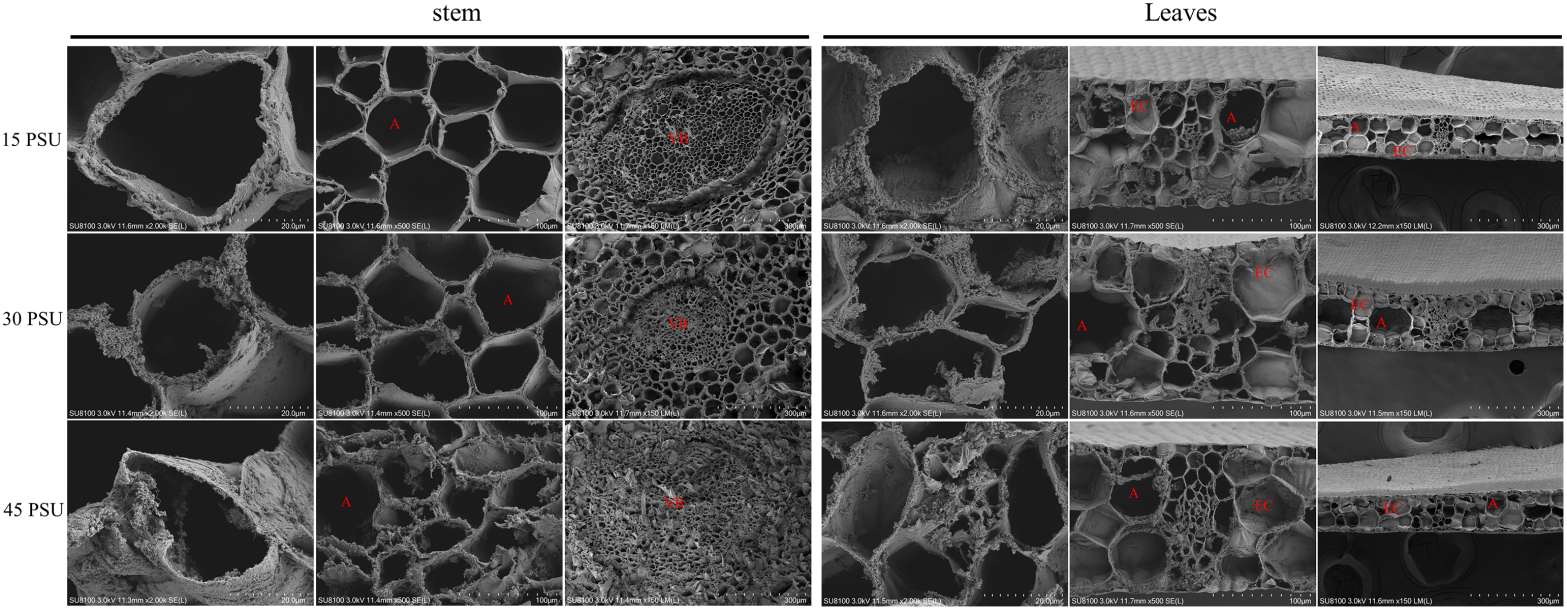
Comparative analysis of the internal structure of stems and leaves at 15 PSU, 30 PSU, and 45 PSU salinity stress by scanning electron microscope (SEM). (A) Aerenchyma; (VB) Vascular bundle; (EC) Epidemic cell.

The parenchyma cells between the vascular bundle and the epidermis of the *T. hemprichii* stem are filled with parenchyma, forming regularly scattered airways. The diameter of the airways is slightly larger than that of the parenchyma cells. In seawater with 30 PSU salinity, the size of the airways was uniformly distributed in a regular arrangement. A single airway hole was regular and circular, with a clear boundary from the central vascular bundle. Under the treatment with a high salinity of 45 PSU, the airway structure shrank and was damaged, the airway holes were deformed and atrophied, and the cell walls were thickened (**Figure 2** and **Supplementary Figure S2**). Under the treatment with a low salinity of 15 PSU, the airway diameter was smaller, the stomata expanded significantly, and the walls were thinner than those under the treatment of 30 PSU.

**Figure 3:**
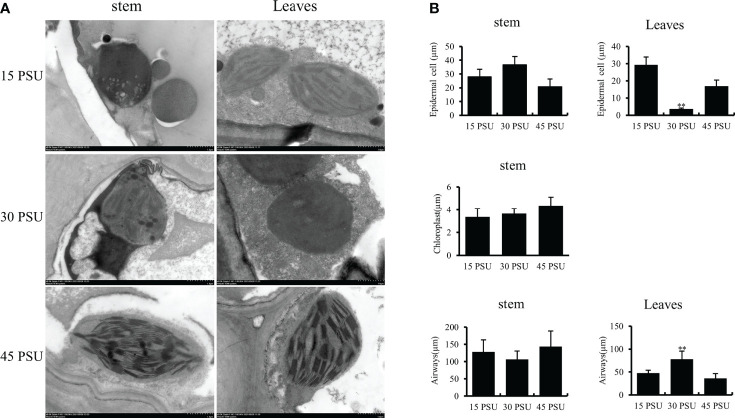
Images of the structure of *T. hemprichi* observed under Transmission electron microscopy (TEM). **(A)** Transmission electron microscopy (TEM) result; **(B)** (EC) Epidemic cell, Chloroplast, and Airways analysis. The statistical significance was assessed using Student’s *t* multiple comparisons test. **P < 0.01.

The microstructure of *T. hemprichii* leaves revealed only one layer of small and dense epidermal cells, which gradually thickened with the increase in salinity. When exposed to high salinity, the proliferative tissue of *T. hemprichii* was prominent, significantly increasing airway diameter. The parenchyma cells increased in size, and the airway shrunk. While the parenchyma cells and the airway were smaller under low salt stress, less proliferative tissues were observed. The epidermal cell wall of *T. hemprichii* leaves was denser and thicker than the cell walls of other parts. Under the treatment with a low salinity of 15 PSU, the degree of epidermal cell wall thickening was less, and the results were similar in the other two treatments (**Figures 2**, **3B**).

The primary functional cells of the stem are epidermal cells, and most of their parenchyma cells contain vacuoles. With a salinity of 30 PSU, the epidermal cells had many organelles, most of them were mitochondria, vacuoles in epidermal cells were small and few. After a low salinity treatment of 15 PSU, the vacuoles in the epidermal cells were significantly increased in quantity and enlarged in volume, and the organelles were concentrated close to the plasma membrane. A 45 PSU treatment caused significant degeneration of the intracellular organelles in epidermal cells, what remains were mainly large vacuoles, and the cell wall thickened significantly. This phenomenon revealed that the effect of hypertonicity was more significant than hypotonic (**Figure 3A**).

*T. hemprichii* cell wall thickened with an increase in salinity. The plasma membrane of epidermal cells was folded, bulged of different sizes and shapes, which is the transmission cell-like structure. The chloroplasts of *T. hemprichii* leaves are mainly concentrated in epidermal cells and mesophyll cytoplasm closing to the epidermal cells. The chloroplasts in epidermal cells of *T. hemprichii* treated with 30 PSU salinity had significantly more numbers than those treated with 15 and 45 PSU salinity. In addition, their shape was plump, kidney-shaped, fusiform, or arcuate; the grana lamellae were neatly arranged; there were a few osmiophilic granules; the structure was compact, and the shape was normal. After low and high salinity treatments, chloroplasts were deformed and distorted; the shape became longer than in 30 PSU, or one end were swollen, irregular, or nearly circular; the grana were irregularly arranged; the lamella spacing increased; the structure were loose; the density was reduced; the number of osmiophilic granules increased (**Supplementary Figure S3**).

### Physiological and biochemical changes under different salinity stresses

#### Soluble protein content

**Figure 4:**
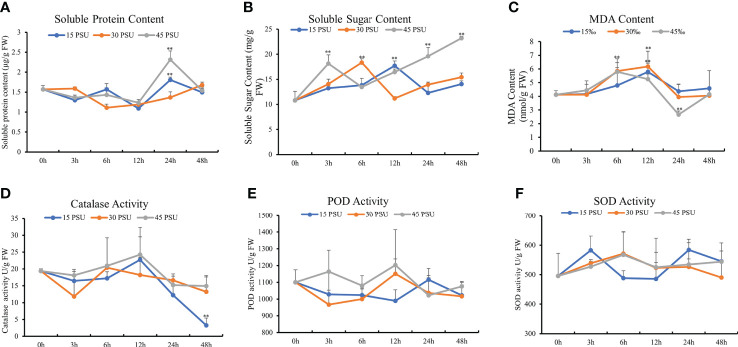
Physiological and biochemical index changes under different salinity conditions. **(A)** Soluble protein content; **(B)** soluble sugar content; **(C)** malondialdehyde (MDA) content; **(D)** catalase activity; **(E)** peroxidase (POD) activity; **(F)** superoxide dismutase (SOD) activity. The statistical significance was assessed using Student’s *t* multiple comparisons test. **P < 0.01.

The soluble protein content of *T. hemprichii* first decreased and then increased under salinity stresses. After the three salinity treatments (15, 30, and 45 PSU), the soluble protein content of *T. hemprichii* was the lowest at 12 hours of stress, reduced by 30.26%, 24.01%, and 21.33% respectively compared with at 0 hours. The soluble protein content under a 45 PSU high salinity treatment was significantly higher than that of a 15 PSU low salinity treatment. With the prolonged stress time, the soluble protein content under 15 and 45 PSU salinity treatments were significantly higher than at 0 hours (*P*<0.01). The soluble protein content under a 45 PSU high salinity treatment was significantly higher than that with a 15 PSU low salinity treatment (**Figure 4A**). High salinity stress rapidly increased the osmotic regulation ability of *T. hemprichii* by increasing soluble protein after stress to reduce salt damage.

### Soluble sugar content

Organic osmotic protective substances, such as proline, soluble sugar, and soluble protein, can maintain the cellular osmotic potential under salinity stress and prevent dehydration. In addition, they play an important role on stabilizing and protecting the structure and function of biological macromolecules. As an intermediate product of energy metabolism in cells, soluble sugar maintains the stability of intracellular pH and ion balance and is a critical osmotic regulator of plant salinity stress. The soluble sugar content of *T. hemprichii* was increased under the three salinity treatments of 15, 30, and 45 PSU. The soluble sugar content increased significantly under a 15 PSU salinity treatment for 6 hours than 0 hours (*P*<0.05). The maximum soluble sugar content was at 12 hours, which was 64.17% higher than at 0 hours. The soluble sugar content under treatments with the salinity of 30 and 45 PSU for 3 hours was significantly higher than that at 0 hours (*P*<0.01), and the maximum soluble sugar content was at 6 hours and 48 hours, 69.84% and 115.04% higher than at 0 hours respectively. The soluble sugar content under 45 PSU salinity treatment for 3 hours was significantly higher than that under treatments with the salinity of 15 and 30 PSU, revealing a significant difference under 24 hours and 48 hours stress compared to the salinity of 15 and 30 PSU treatments (**Figure 4B**). The soluble sugar content of *T. hemprichii* was high under prolonged stress time. High salinity treatment significantly increased soluble sugar content, indicating that *T. hemprichii* had strong salt tolerance. Therefore, high salinity stress promotes the accumulation of soluble sugars.

### MDA content

The MDA content reflects the degree of damage to plants under salinity stress. The MDA content of *T. hemprichii* was significantly higher under 30 and 45 PSU treatments for 6 and 12 hours than at 0 hours. Compared with the control group, the MDA content of *T. hemprichii* treated with 30 and 45 PSU salinity for 6 hours increased by 42.01% and 40.61%, respectively. while the MDA content of *T. hemprichii* treated with 30 and 45 PSU salinity for 12 hours increased by 49.97% and 27.72%respectively. The MDA content under treatments with higher salinity of 30 and 45 PSU for 6 hours increased by 21.95% and 20.74%, compared with that under treatment with a salinity of 15 PSU. Therefore, salinity stress damaged the biofilm, causing membrane lipid peroxidation and increasing MDA content significantly. With the prolonged stress time, the MDA content in *T. hemprichii* significantly reduced under treatment with a salinity of 45 PSU for 24 hours (**Figure 4C**). This could be caused by the plant resistance mechanisms of the high salinity stress. The reduction of MDA content affected membrane lipid peroxidation, protected the cell membrane system and alleviated the effects of salinity stress on plants. We also detected the activities of CAT, POD, and SOD. However, no significant difference under different salinity stresses was observed (**Figures 4D-F**).

### Gene expression variations under different salinity stresses

#### Sequencing statistics

A total of 47 samples were measured using the DNBSEQ platform, producing an average of 21.49 M data each (**Supplementary Table 1**). The clean reads were aligned to the reference gene sequence using Bowtie2. A total of 190,908 transcripts were detected, and the ratio of mapping to the reference sequence (unpublished) was 73.01% ~ 91.91%. The average mapped ratio was 80.24% (**Supplementary Table 2**). The overall transcriptome sequencing analysis, including randomness, coverage, and sequencing saturation statistics, revealed that the sequencing results were reliable. The lengths of the obtained transcripts were more than 300 bp, 108,002 of which were between 300 and 1000 bp, accounting for 56.57% of the total transcripts (**Supplementary Table S3**). The correlation analysis revealed a high correlation between samples, the result consistented with the gene expression level (**Supplementary Figure S4**). Also, the expression distribution of each sample presented a normal distribution (**Supplementary Figure S5**). The assembled sequence data for these raw reads were deposited at the NCBI Sequence Read Archive (SRA, http://www.ncbi.nlm.nih.gov/Traces/sra) under the accession number PRJNA848129.

### Temporal characteristics under different salinity stresses

**Figure 5:**
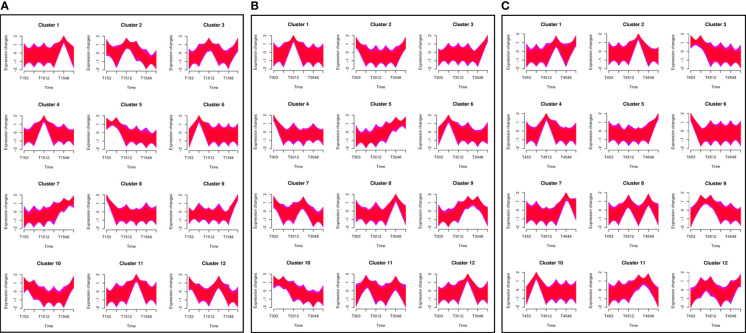
Clustering analysis of differentially expressed genes (DEGs) and time-series analysis of DEGs under different salinity conditions. A total of 108,002 unigenes were classified into 12 clusters using the Mfuzz package in R. A. The horizontal axis shows the different groups, while the vertical axis shows the time series of the gene expression levels. **(A)** Under a 15 PSU treatment; **(B)** Under a 30 PSU treatment; **(C) **Under a 45 PSU treatment.

The clustering analysis of 108,002 unigenes was performed across 45 samples to define the temporal characteristics of the complete transcriptome dataset by Mfuzz, and all the unigenes were divided into 12 clusters. Different salinity treatments greatly affected the gene expression pattern of *T. hemprichii*. Under the low salinity treatment of 15 PSU, the gene expression pattern varied gently (**Figure 5A**, clusters 5, 7, 8, 9, 10) or greatly at one of the time points (**Figure 5A**, clusters 1, 2, 3, 4, 6, 11, 12). Compared with the treatment of 30 PSU, no clusters with large variations in gene expression were observed (**Figure 5B**, clusters 9, 11). We observed more variation patterns and more intense variation in gene differential expression patterns under a high salinity treatment of 45 PSU. The numbers of intense variation clusters under a 45 PSU treatment were significantly more than under a 30 PSU treatment (**Figure 5C**, cluster 8,9,11,12). Therefore, hypertonicity significantly alters gene expression patterns, while hypotonicity is weaker than hypertonicity.

### Differential expression analysis

Compared with the control group, both the 45 PSU and 15 PSU treatments had up-regulated genes significantly more than the down-regulated genes (**Supplementary Figure S6A**). The DEGs under 15 PSU and 45 PSU treatments of *T. hemprichii* were significantly more than under 30 PSU treatments for 3 and 6 hours. Then, the number of DEGs gradually decreased to the same level as the treatment with a salinity of 30 PSU (**Supplementary Figure S6B**). From the proportion of DEGs, up-regulated genes were more than down-regulated genes. Regardless of high salinity or low salinity stress, the up-regulated genes were treble down-regulated genes after 3 hours of treatments. After 6 hours of treatments, the ratio of up- and down-regulated genes under low salinity treatment increased, while the ratios of up- and down-regulated genes were similar under both 30 PSU and 45 PSU treatments. Subsequently, the ratio of up- and down-regulated genes under 15 PSU treatment was equivalent to that under 30 PSU treatment. However, the ratio of up- and down-regulated genes under the high salinity treatment fluctuated periodically at 24 hours, which may be related to the growth rhythm of *T. hemprichii* under hypertonic conditions (**Supplementary Figure S6C**).

**Figure 6:**
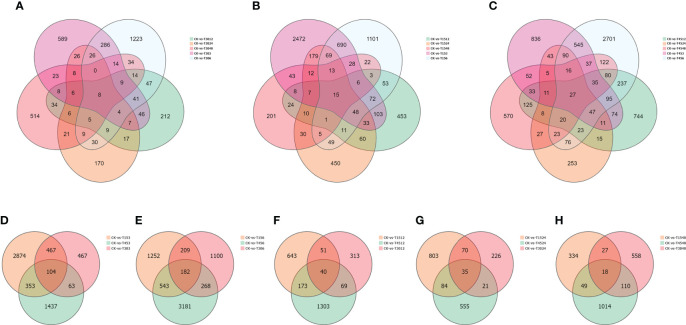
Differentially expressed genes (DEGs) under different treatments conditions. **(A)** Comparative analysis of DEGs under the same salinity condition (15 PSU) and different time conditions (3, 6, 12, 24, and 48 hours); **(B)** Comparative analysis of DEGs under the same salinity condition(30 PSU) and different time conditions (3, 6, 12, 24, 48 hours); **(C)** Comparative analysis of DEGs under the same salinity condition(45 PSU) and different time conditions (3, 6, 12, 24, 48 hours); **(D)** Comparative analysis of DEGs under different salinities after 3 hour treatment; **(E)** Comparative analysis of DEGs under different salinities after 6 hour treatment; **(F)** Comparative analysis of DEGs under different salinities after 12 hour treatment; **(G)** Comparative analysis of DEGs under different salinities after 24 hour treatment; **(H)** Comparative analysis of DEGs under different salinities after 48 hour treatment.

Subsequently, DGEs were analyzed. Only eight co-expressed genes were present at each time point under treatment with a salinity of 30 PSU (**Figure 6A**; **Supplementary Table 4**). The co-expressed genes were probable *methyltransferase*, *putative glucose-6-phosphate 1-epimerase*, *putative bystin*, *cytochrome c1-2*, *protein stay-green-like chloroplastic*, *protein LHCP translocation defect*, *MLP-like protein 423* and *mitochondrial inner membrane protein mitofilin*. At each time point under 15 PSU treatment, the specifically expressed genes were the majority. A total of 690 genes were co-expressed after 3 and 6 hours of treatments, and only 15 unigenes were expressed at each time point (**Figure 6B**; **Supplementary Table S4**). A lot of genes were specifically expressed at each time point under a high salinity treatment of 45 PSU, and rhythmic changes appeared with prolonged time. A total of 27 co-expressed genes were observed at six-time points (**Figure 6C**; **Supplementary Table S5**), significantly more than under the low and normal salinity treatments. Commonly expressed genes under the three salinity treatments were totally different, but most of them were associated with salinity stress.

**Figure 7:**
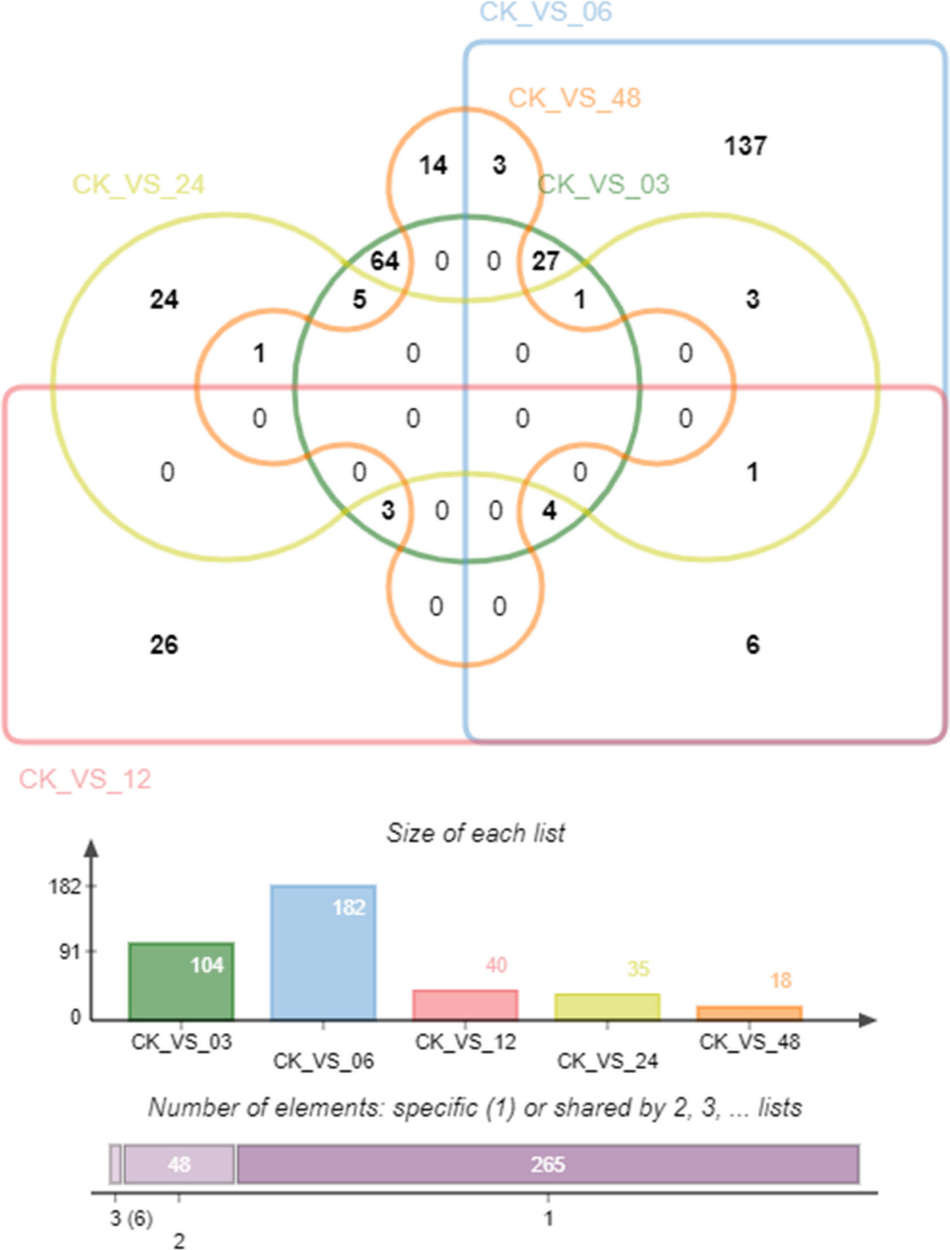
Distribution of differentially expressed genes (DEGs) at different time points.

**Figure 8:**
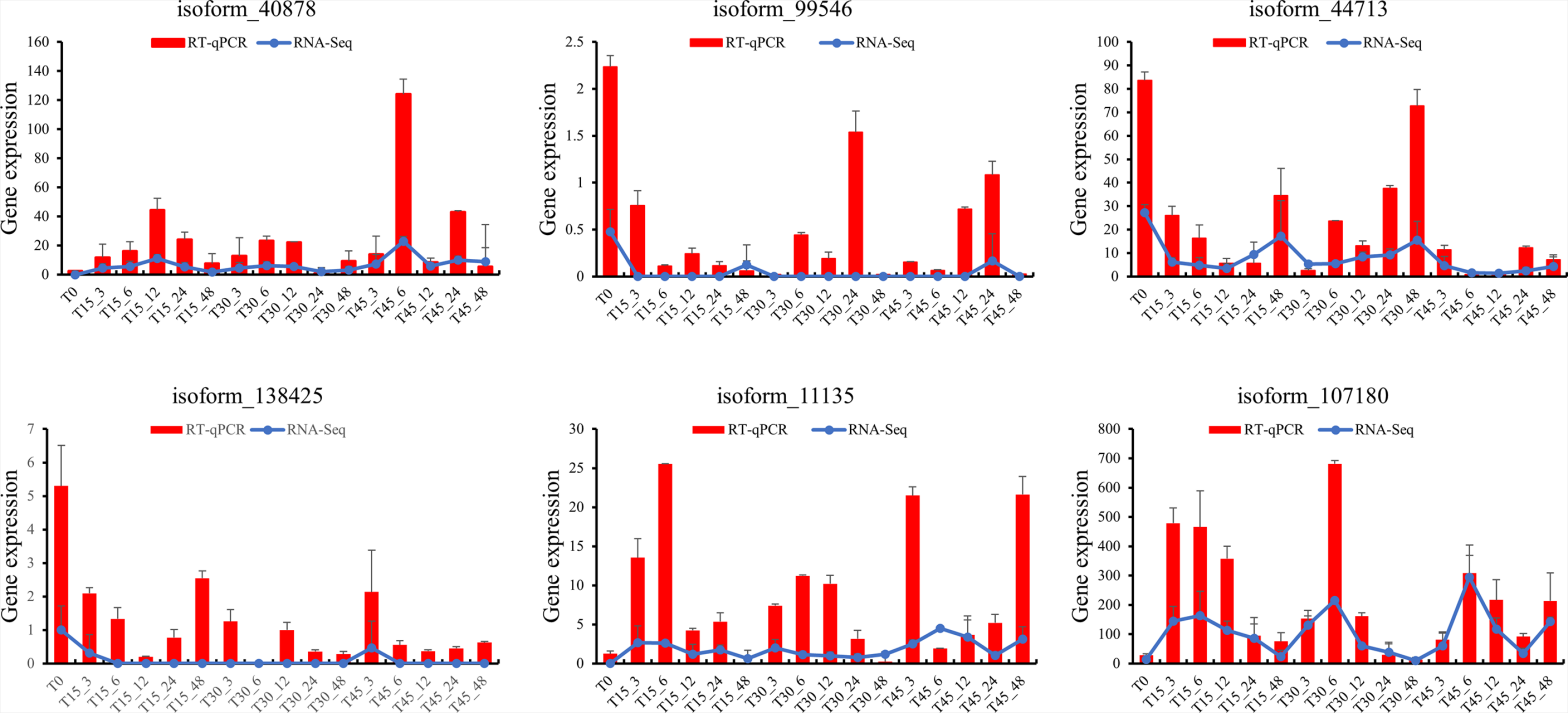
Comparative analysis of the quantitative polymerase chain reaction (qPCR) and transcriptome data for six genes.

We analyzed co-expressed genes under different salinity stresses at the same time point by the same method. The co-expressed genes were up to 182 after 6 hours of treatments and then gradually decreased at 12 hours (40), 24 hours (35), and 48 hours (18) (**Figures 6D–H**). As salinity treatments of 15 PSU and 30 PSU, the genes in *T. hemprichii* varied rapidly, and gradually formed a stable tolerance mechanism over time, while the gene varied drastically all the time under high salinity stress. Subsequently, we performed a Venn diagram analysis of these co-expressed genes. No co-expressed genes at different time points were observed. Only six genes were co-expressed at three-time points: isoform_44713 (*cytochrome b561* and *DOMON domain-containing protein*), isoform_99546 (*mitochondrial inner membrane protein*), isoform_40878 (no annotation), isoform_107180 (*protein gigantea-like*), isoform_11135 (*probable methyltransferase*), and isoform_138425 (*putative bystin*) (**Figure 7**). Combined with quantitative reverse-transcription PCR (RT-qPCR) technology, we detected the expression patterns of the six genes at different time points under treatment with salinity of 30 PSU. The results were consistent with the transcriptome sequencing (**Figure 8**).

KEGG and GO cluster analyses were performed on the 319 genes. The genes involving in stress response were enriched in KEGG and GO classifications (**Supplementary Figure S7**). From the KEGG pathway, the DEGs at all stress time points were enriched in six pathways: “transport and catabolism”, “carbohydrate metabolism”, “global and overview maps”, “biosynthesis of other secondary”, “metabolites”, and “environmental adaptation”. From GO enrichment analysis, the DEGs at all stress time points were enriched in ten pathways: “cellular anatomical entity”, “intracellular binding”, “transcription regulator activity”, “cellular process”, “metabolic process”, “catalytic activity”, “localization”, “protein-containing complex” and “biological regulation”. Although these common DEGs varied widely among these time points, the enrichment classification was very concentrated, suggesting that these pathways may be key factors in salt tolerance.

## Discussion

Salinity stress is a key factor that affects the healthy growth of plants by inhibiting photosynthesis (Munns, 1993). Plant tolerance to salinity promotes its growth in adverse environments. For example, the marine angiosperm *Zostera marina* had a lower survival rate under varying salinity (Johnson et al., 2021). However, marine plants are more tolerant of salinity than terrestrial plants. The optimum salinity range for Thalassia is 24 to 35 PSU (Phillips, 1960; McMillan and Moseley, 1967; Zieman, 1975). However, it tolerates a broad salinity range for very brief exposures, ranging from 3.5 to 60 PSU (McMillan and Moseley, 1967). Nevertheless, such exposures commonly result in leaf loss.

*In situ* salinity of the seawater in the seagrass bed of Xincun Bay was approximately 30 PSU (Yang and Yang, 2009), acting as control levels in the laboratory experiments. In this study, the salinity of 25 to 35 PSU promoted the growth potential of *T. hemprichii* under treatments after 28 days, the survival rate was between 81.22% and 93.77%. On the other hand, the survival rate of *T. hemprichii* was 35.35% when the salinity was 50 PSU, and the survival rate was significantly affected under low salinity stress, showing that *T. hemprichii* could grow in a wide range of salinity. Similar conclusions were foundin other aquatic plants. For example, the optimal salinity for the growthof *Halophia baccarii* is 25 to 30 PSU, however, the length and width of the leaves are significantly shorter and narrower under 0.52 to 4 PSU and45 PSU salinity stress (Fakhrulddin et al., 2013). Under 30 PSU and sufficient light conditions, *Halophila ovalis* grows better than in other salinity conditions (Bujang et al., 2008; Ralph, 1998).

Microstructural results showed that, like other marine plants, *T. hemprichii* had many dissociative parenchyma cells, or the whole cells collapsed into cavities and air cells, forming many airways. The airways and parenchyma cells in *T. hemprichii* showed significant structural variation with the salinity changes (Watkin et al., 1998). The effect of high salinity on the structure of airways were significantly higher than that of low salinity, indicating that salinity is one of the key factors affecting the structure of airways. High salinity thickened hyperplasia tissues around the stem of *T. hemprichii*, similar structures were found on the leaves surface. Several organelles were observed in the epidermal cells of stems, such as mitochondria. Changes in mitochondrial morphology reflect the tolerance to salinity stress in plants, the structures of mitochondrial is destroyed under high salinity stress in sensitive cells (Domondon et al., 2020; Munns et al., 2020). The volume of the mitochondrial expanded and the structure were significantly damaged under 45 PSU stress, while the mitochondrial structure were relatively stable under 30 PSU stress. Therefore, the stems and leaves of *T. hemprichii* absorbed and accumulated a higher concentration of inorganic ions from the vacuoles of the parenchyma cells, reducing the water potential and adjusting the osmotic pressure. A lot of airways and parenchyma cells in the stem disintegrated with the prolonged time under 45 PSU high salinity stress, and the functional cells of the epidermis were damaged.

Plant stems under salinity stress reduce water loss by thickening the cuticle, epidermis, and mechanical tissue (Stelzer and Luchli, 1978). The microstructure revealed that, due to high salinity, epidermal cell wall of *T. hemprichii* leaves thickened. Chloroplast is one of the most salinity-sensitive organelles in plant cells (Chen et al., 2021), and the chloroplast ultrastructure is closely related to salt tolerance (Wu et al., 2021; Zhu et al., 2021). Many halophyte leaves have osmiophilic granules, which can reduce the osmotic potential, increase the cytoplasmic concentration, and ensure the absorption of water and inorganic salts (Mamane and Gottlieb, 1992; He et al., 2022). The increase of osmiophilic granules is one of the biomarkers for plant salt tolerance. Many osmiophilic granules were found in *T. hemprichii* under different salinity stresses, indicated that osmiophilic granules were ubiquitous in *T. hemprichii* cells. The chloroplast structure was relatively intact, with fewer osmiophilic granules under 30 PSU salinity stress, indicating that optimal salinity conditions are required for the normal growth of *T. hemprichii*.Organic osmotic protective substances, such as proline, soluble sugar, and soluble protein, maintain osmotic potential of plant cell to prevent dehydration under salinity stress. Thus, they stabilize and protect the structure and function of biological macromolecules. For example, high salinity stress damaged corn seedlings, reduced their regulation ability, the soluble sugar and protein content first increased and then decreased with the increase of salinity (Huang et al., 2022). Tomato seedlings subjected to salinity stress revealed similar results (Chaudhary et al., 2020; Ali et al., 2021). *T. hemprichii* is a marine plant and different from terrestrial plants. The content of soluble sugar and soluble protein increased with a long-term salinity stress, especially under a high salinity stress.The MDA content reducing significantly may avoid oxidative damage, which is an important physiological indicator of plant resistance to salinity stress. The MDA content of *T. hemprichii* was high after the prolonged treatment under salinity stress, increasing by 49.97% after 12 hours treatment. However, the MDA content decreased significantly after 24 hours of high salinity treatment, which may be due to the generation of the resistance mechanism of *T. hemprichii* under high salinity stress. The low MDA content reduced the lipid peroxidation of plant membranes, protected the cell membrane system of plants, and alleviated salinity stress effects on plants in the degree. Different to terrestrial plants, the POD, SOD, and CAT contents remained almost unchanged in *T. hemprichii* under different salinity stresses. High salinity stress promoted the rapid accumulation of soluble sugar and soluble protein. Furthermore, the decrease of MDA content in *T. hemprichii* reduced the lipid peroxidation of plant membranes, formed the high adaptability of *T. hemprichii* to a wide range of salinity changes.

The molecular mechanisms underlying the response of plants to salinity stress are complex. Many studies have focused on the changes in gene expression in terrestrial plants after salinity stress (Chanwala et al., 2020; Yuan et al., 2021). However, few studies have been conducted on marine plants. In this study, the typical marine plant *T. hemprichii* was treated with different salinity levels, and its early gene response patterns were analyzed. After salinity treatments, *T. hemprichii* had fewer DEGs in overall number. The pathways in response to high salinity environments of terrestrial plants could be divided into abscisic acid-responsive salinity stress pathways and abscisic acid-independent salinity stress pathways (Punia et al., 2021). In this study, the differential genes of *T. hemprichii* were not enriched in the abscisic acid signaling pathway, but concentrated in the pathways associated with transportation, metabolism, and environmental adaptation after treatments. The commonality analysis of DEGs at each time point was also in line with the results. Among the 319 commonly expressed DGEs, no single gene was consistently differentially expressed at different concentrations and time points. The changes of gene expression in *T. hemprichii* were sharp in the early stage after suffering from salinity stress. From the time-series analysis, the variations of gene expression were drastically after 3 and 6 hours of treatments, while changes were more intense with high salinity stress.

Herein, we studied the changes in physiological characteristics and microstructure of the shoal marine plant *T. hemprichii* under different salinity stresses. The comparative transcriptome analysis under different salinity stresses was also analyzed. This is the first systematic study on the response of *T. hemprichii* to different salinity stresses. This study revealed that *T. hemprichii* had a high fitness to salinity, with more than 35% survival rate under 50 PSU treatment for 28 days. However, under the high and low salinity conditions, the growth and development of *T. hemprichii* showed of retardation and yellowing in different degrees. Meanwhile, the parenchyma cells in *T. hemprichii* collapsed, the contents of soluble sugar, soluble protein and MDA changed, indicating that *T. hemprichii* was sensitive to salinity variation. The growth and development of *T. hemprichii* were significantly affected, although without plant death. From the transcriptome results, its molecular characteristics were different from the salt tolerance mechanism of terrestrial plants. This study provided a basis for a deep study on the mechanism underlying the response of *T. hemprichii* to shoal living environment changes and data support for the study of the molecular mechanism of *T. hemprichii* in response to salinity stress.

## Data availability statement

The original contributions presented in the study are publicly available. This data can be found here: NCBI, PRJNA848129.

## Author contributions

JS, DW designed the experiments. JS, ZW, and LY analyzed the data. SC and ZC collected the samples. XG collected the Electron microscope picture. JS, DW wrote the paper. All authors contributed to the article and approved the submitted version.

## Funding

This work was supported by Major public welfare scientific research project of Hainan Province Science and Technology Special Fund (ZDYF2020202), Fund of Department budget projects of Hainan Province in 2022 (KYL-2022-07), National Natural Science Foundation of China (42166006), Hainan Provincial Basic and Applied Basic Research Fund for High-Level Talents in Natural Science (421RC662), Fund of Key Laboratory of Marine Ecological Conservation and Restoration, Ministry of Natural Resources/Fujian Provincial Key Laboratory of Marine Ecological Conservation and Restoration (EPR2022005). The funders had no role in study design, data collection and analysis, decision to publish, or preparation of the manuscript.

## Conflict of interest

The authors declare that the research was conducted in the absence of any commercial or financial relationships that could be construed as a potential conflict of interest.

## Publisher’s note

All claims expressed in this article are solely those of the authors and do not necessarily represent those of their affiliated organizations, or those of the publisher, the editors and the reviewers. Any product that may be evaluated in this article, or claim that may be made by its manufacturer, is not guaranteed or endorsed by the publisher.

## References

[B1] Ali M. M. Jeddi K. Attia M. S. Elsayed S. M. Yusuf M. Osman M. S.. (2021). Wuxal amino (Bio stimulant) improved growth and physiological performance of tomato plants under salinity stress through adaptive mechanisms and antioxidant potential. Saudi J. Biol. Sci. 28, 3204–3213. doi: 10.1016/j.sjbs.2021.04.040 34121857 PMC8176060

[B2] Bandeira S. Gell F. (2003). “The seagrasses of Mozambique and southeastern Africa,” in World atlas of seagrasses. Eds. Green E. Short F. (Berkeley: University of California Press), 93–100.

[B3] Bujang J. S. Huat L. L. Zakaria M. H. Arshad A. Ogawa H. (2008). Laboratory culture of the seagrass, *halophila ovalis* (R.Br.) hooker f. Mar. Res. Indonesia 33 (1), 1–6. doi: 10.14203/mri.v33i1.500

[B4] Bustin S. A. Benes V. Garson J. A. Hellemans J. Huggett J. Kubista M.. (2009). The MIQE guidelines: Minimum information for publication of quantitative real-time PCR experiments. American Association for Clinical Chemistry 55 (4), 611–622. doi: 10.1373/CLINCHEM.2008.112797 19246619

[B5] Campbell S. McKenzie L. (2004). Flood related loss and recovery of intertidal seagrass meadows in southern Queensland, Australia. Estuarine Coast. Shelf Sci. 60, 477–490. doi: 10.1016/j.ecss.2004.02.007

[B6] Cao L. Wang W. Yang Y. Yang C. Yuan Z. Xiong S.. (2007). Environmental impact of aquaculture and countermeasures to aquaculture pollution in China. Environ. Sci. pollut. Res. 14 (7), 452–462. doi: 10.1065/espr2007.05.426 18062476

[B7] Chanwala J. Satpati S. Dixit A. Parida A. Giri M. K. Dey N. (2020). Genome-wide identification and expression analysis of WRKY transcription factors in pearl millet (*Pennisetum glaucum*) under dehydration and salinity stress. BMC Genomics 21, 231. doi: 10.1186/s12864-020-6622-0 32171257 PMC7071642

[B8] Chaudhary K. Kumar S. Patil S. (2020). Alleviation of salt stress in alleviation of salt stress in solanaceous solanaceous vegetables through halo priming. Available at: https://researchgate.net/publlcation/344783336.

[B9] Chen G. Y. He X. Priyadarshani S. Wang Y. Qin Y. (2021). Assembly and comparative analysis of the complete mitochondrial genome of *suaeda glauca* . BMC Genomics 22, 167. doi: 10.1186/s12864-021-07490-9 33750312 PMC7941912

[B10] Chollett I. Bone D. Pérez D. (2007). Effects of heavy rainfall on *Thalassia testudinum* beds. Aquat. Botany 87, 189–195. doi: 10.1016/j.aquabot.2007.05.003

[B11] Den H. (1970). The sea-grasses of the world (North Holland Publ. Co., Amsterdam).

[B12] Deng W. H. Lü X. F. (2018). Advances in studies on salt-tolerance mechanism of *zostera marina* . Plant Physiol. J. 54, 718–724. doi: 10.13592/j.cnki.ppj.2017.0425

[B13] Domondon M. Polina I. Nikiforova A. B. Sultanova R. F. Kruger C. Vasileva V. Y.. (2020). Renal glomerular mitochondria function in salt-sensitive hypertension. Front. Physiol. 10 (10), 1588. doi: 10.3389/fphys.2019.01588 32116733 PMC7010849

[B14] Duarte C. M. Marbà N. Gacia E. Fourqurean J. W. Beggins J. Barrón C.. (2010). Seagrass community metabolism: Assessing the carbon sink capacity of seagrass meadows. Global Biogeochemical Cycles 24, GB4032. doi: 10.1029/2010GB003793

[B15] Edgar G. J. Shaw C. Watsona G. F. Hammond L. S. (1994). Comparisons of species richness, size-structure and production of benthos in vegetated and unvegetated habitats in Western port, Victoria. J. Exp. Mar. Biol. Ecol. 176, 201–226. doi: 10.1016/0022-0981(94)90185-6

[B16] Fakhrulddin L. M. Sidik B. J. Harah Z. M. (2013). *Halophila beccarii* aschers. (Hydrocharitaceae) responses to different salinity gradient. J. Fisheries Aquat. Sci. 8 (3), 462–471. doi: 10.3923/jfas.2013.462.471

[B17] Faxneld S. Jörgensen T. L. Tedengren M. (2010). Effects of elevated water temperature, reduced salinity and nutrient enrichment on the metabolism of the coral turbinaria mesenterina. Estuarine Coast. Shelf Sci. 88 (4), 482–487. doi: 10.1016/j.ecss.2010.05.008

[B18] Funge-Smith S. J. Briggs M. R. P. (1998). Nutrient budgets in intensive shrimp ponds: implications for sustainability. Aquaculture 164 (1), 117–133. doi: 10.1016/S0044-8486(98)00181-1

[B19] Heil L. (2000). Impact of shrimp farming on mangroves along india's east coast. Unasylva 203 (512), 48–55. doi: 10.1016/j.gloenvcha.2018.05.005

[B20] Hemminga M. A. Duarte C. M. (2000). Seagrass ecology (Cambridge: University Press).

[B21] He L. Su R. Chen Y. Zeng P. Du L. Cai B.. (2022). Integration of manganese accumulation, subcellular distribution, chemical forms, and physiological responses to understand manganese tolerance in *macleaya cordata* . Environ. Sci. pollut. Res. 29, 39017–39026. doi: 10.1007/s11356-022-19562-8 35306649

[B22] Huang X. Huang L. Li Y. Xu Z. Fong C. W. Huang D.. (2006). Main seagrass beds and threats to their habitats in the coastal sea of south China. Chin. Sci. Bulletin 51, 114–119. doi: 10.1007/s11434-006-9136-5

[B23] Huang X. P. Jiang Z. J. Zhang J. P. Yu S. Liu S. Wu Y. (2018). The Chinese nomenclature of the global seagrasses. Haiyang Xuebao 40, 127–133. doi: 10.3969/j.issn.0253-4193.2018.04.012

[B24] Huang C. Zhang W. Wang H. Gao Y. Ma S. Qin A.. (2022). Effects of waterlogging at different stages on growth and ear quality of waxy maize. J. Agric. Water Management 266, issue C. doi: 10.1016/j.agwat.2022.107603

[B25] IPCC. (2007). Climate change 2007: the physical science basis (Cambridge University Press).

[B26] Jahnke M. Gullstrom M. Larsson J. Asplund M. E. Mgeleka S. Silas M. O.. (2019). Population genetic structure and connectivity of the seagrass *Thalassia hemprichii* in the Western Indian ocean is influenced by predominant ocean currents. Ecol. And Evol. 9, 8953–8964. doi: 10.1002/ece3.5420 PMC6706205 31462994

[B27] Jiang Z. Huang X. Zhang J. (2013). Effect of nitrate enrichment and salinity reduction on the seagrass *Thalassia hemprichii* previously grown in low light. J. Exp. Mar. Biol. Ecol. 443, 114–122. doi: 10.1016/j.jembe.2013.02.034

[B28] Johnson A. J. Shields E. C. Kendrick G. A. Orth R. J. (2021). Recovery dynamics of the seagrass *Zostera marina* following mass mortalities from two extreme climatic events. Estuaries Coasts 44, 535–544. doi: 10.1007/s12237-020-00816-y

[B29] Larkum A. Orth R. J. Duarte C. M. (2006). Seagrasses: Biology, ecology and conservation: Seagrasses: Biology, ecology and conservation. (Springer)

[B30] Lee C. L. Huang Y. H. Chen C. H. Lin H. J. (2016). Remote underwater video reveals grazing preferences and drift export in multispecies seagrass beds. J. Exp. Mar. Biol. Ecol. 476, 1–7. doi: 10.1016/j.jembe.2015.12.004

[B31] Livak K. J. Schmittgen T. D. (2001). Analysis of relative gene expression data using real-time quantitative PCR and the 2(-Delta Delta C(T)) method. Methods 25, 402–408. doi: 10.1006/meth.2001.1262 11846609

[B32] Mamane Y. Gottlieb J. (1992). Nitrate formation on sea-salt and mineral particles–a single particle approach. Atmospheric Environ. Part A Gen. Topics 26, 1763–1769. doi: 10.1016/0960-1686(92)90073-T

[B33] McMillan C. Moseley F. N. (1967). Salinity tolerances of five marine spermatophytes of redfish bay, Texas. ecology 18, 503–506. doi: 10.2307/1932688

[B34] Munns R. (1993). Physiological processes limiting plant growth in saline soil: some dogmas and hypotheses. Plant Cell Environ 16, 15–24. doi: 10.1111/j.1365-3040.1993.tb00840.x

[B35] Munns R. (2002). Comparative physiology of salt and water stress.pdf. Plant Cell Environment 25, 239–250. doi: 10.1046/j.0016-8025.2001.00808.x 11841667

[B36] Munns R. Day D. A. Fricke W. Watt M. Arsova B. Barkla B. J.. (2020). Energy costs of salt tolerance in crop plants. New Phytologist 225, 1072–1090. doi: 10.1111/nph.15864 31004496

[B37] Nordlund L. M. Koch E. W. Barbier E. B. Creed J. C. (2017). Seagrass ecosystem services and their variability across genera and geographical regions. PloS One 12, e0163091. doi: 10.1371/journal.pone.0169942 PMC5061329 27732600

[B38] Orth R. J. Carruthers T. J. B. Dennison W. C. Duarte C. M. Fourqurean J. W. Heck K. L.. (2006). A global crisis for seagrass ecosystems. Bioscience 56, 987–996. doi: 10.1641/0006-3568(2006)56[987:AGCFSE]2.0.CO;2

[B39] Phillips R. C. (1960). Observations on the ecology and distribution of the Florida seagrasses. (Florida State Board of Conservation, Marine Laboratory)

[B40] Phillips R. C. Meñez E. G. (1988). Seagrasses (Smithsonian Institution Press).

[B41] Punia H. Tokas J. Malik A. Sangwan S. Rani A. Yashveer S.. (2021). Genome-wide transcriptome profiling, characterization, and functional identification of NAC transcription factors in sorghum under salt stress. Antioxidants-Basel 10, 1605. doi: 10.3390/antiox10101605 34679740 PMC8533442

[B42] Ralph P. J. (1998). Photosynthetic responses of *halophila ovalis* (r. br.) hook. f. to osmotic stress. Journal of Experimental Marine Biology & Ecology 227 (2), 203–220. doi: 10.1016/S0022-0981(97)00269-4

[B43] Shen J. Cai Z. Chen S. Wang D. Wu Z. (2021). Transcriptome-wide identification and characterization of the MYB gene family in sickle seagrass (*Thalassia hemprichii*). Ecol. Genet. Genomics 20, 100093. doi: 10.1016/j.egg.2021.100093

[B44] Sinclair E. A. Anthony J. M. Greer D. Ruiz-Montoya L. Evans S. M. Krauss S. L.. (2016). Genetic signatures of bassian glacial refugia and contemporary connectivity in a marine foundation species. J. Biogeogr. 43, 2209–2222. doi: 10.1111/jbi.12822

[B45] Spalding M. D. Taylor M. L. Ravilious C. Short F. Green E. (2003). The distribution and status of seagrasses (University of California Press).

[B46] Stelzer R. Luchli A. (1978). Salt- and flooding tolerance of puccinellia peisonis III. distribution and localization of ions in the plant. Z. für Pflanzenphysiologie 88, 437–448. doi: 10.1016/S0044-328X(78)80260-8

[B47] Touchette B. W. (2007). Seagrass-salinity interactions: Physiological mechanisms used by submersed marine angiosperms for a life at sea. J. Exp. Mar. Biol. Ecol. 350, 194–215. doi: 10.1016/j.jembe.2007.05.037

[B48] Wainwright B. J. Arlyza I. S. Karl S. A. (2018). Population genetic subdivision of seagrasses, *syringodium isoetifolium* and *Thalassia hemprichii*, in the Indonesian archipelago. Bot. Mar. 61, 235–245. doi: 10.1515/bot-2017-0058

[B49] Watkin E. L. J. Thomson C. J. Hank G. (1998). Root development and aerenchyma formation in two wheat cultivars and one triticale cultivar grown in stagnant agar and aerated nutrient solution. Ann. Bo. 81 (2), 349–354. doi: 10.1006/anbo.1997.0565

[B50] Waycott M. Collier C. McMahon K. Ralph P. McKenzie L. Udy J.. (2007). “Vulnerability of seagrasses in the great barrier reef to climate change,” in Climate change and the great barrier reef great barrier reef. Eds. Johnson J. E. Marshall P. A. (Australia: Great Barrier Marine Park Authority and Australian Greenhouse Office), 193–235.

[B51] Waycott M. Duarte C. M. Carruthers T. J. B. Orth R. J. Dennison W. C. Olyarnik S.. (2009). Accelerating loss of seagrasses across the globe threatens coastal ecosystems. Proceeding Natl. Acad. Sci. United States America 106, 12377–12381. doi: 10.1073/pnas.0905520106 PMC2707273 19587236

[B52] Williams J. A. Holt G. J. Robillard M. M. R. Holt S. A. Hensgen G. Stunz G. W. (2016). Seagrass fragmentation impacts recruitment dynamics of estuarine-dependent fish. J. Exp. Mar. Biol. Ecol. 479, 97–105. doi: 10.1016/j.jembe.2016.03.008

[B53] Wissler L. Codoñer F. M. Gu J. Reusch T. B. H. Olsen J. L. Procaccini G.. (2011). Back to the sea twice: identifying candidate plant genes for molecular evolution to marine life. BMC Evolutionary Biol. 11 (1), 8. doi: 10.1186/1471-2148-11-8 PMC3033329 21226908

[B54] Wu C. Cao S. Xie K. Chi Z. Wang J. Wang H.. (2021). Melatonin delays yellowing of broccoli during storage by regulating chlorophyll catabolism and maintaining chloroplast ultrastructure. Postharvest Biol. Tech. 172, 111378. doi: 10.1016/j.postharvbio.2020.111378

[B55] Yang D. Yang C. (2009). Detection of seagrass distribution changes from 1991 to 2006 in xincun bay, hainan, with satellite remote sensing. Sensors 9, 830–844. doi: 10.3390/s90200830 22399941 PMC3280833

[B56] Yuan H. Guo W. Zhao L. Yu Y. Chen S. Tao L.. (2021). Genome-wide identification and expression analysis of the WRKY transcription factor family in flax (*Linum usitatissimum L.*). BMC Genomics 22, 375. doi: 10.1186/s12864-021-07697-w 34022792 PMC8141250

[B57] Zhu D. Luo F. Zou R. Liu J. Yan Y. (2021). Integrated physiological and chloroplast proteome analysis of wheat seedling leaves under salt and osmotic stresses. J. Proteomics 234, 104097. doi: 10.1016/j.jprot.2020.104097 33401000

[B58] Zieman J. C. (1975). Seasonal variation of turtle grass, *thalassia testudinum* konig, with reference to temperature and salinity effects. Aquat Bot. 1, 107–123. doi: 10.1016/0304-3770(75)90016-9

