# Supplementary figures and images for: Physiological basis and differentially expressed genes in the salt tolerance mechanism of Thalassia hemprichii
Source: Front Plant Sci. 2022 Nov 28;13:975251. doi: 10.3389/fpls.2022.975251 (PMC9742478; doi:10.3389/fpls.2022.975251)

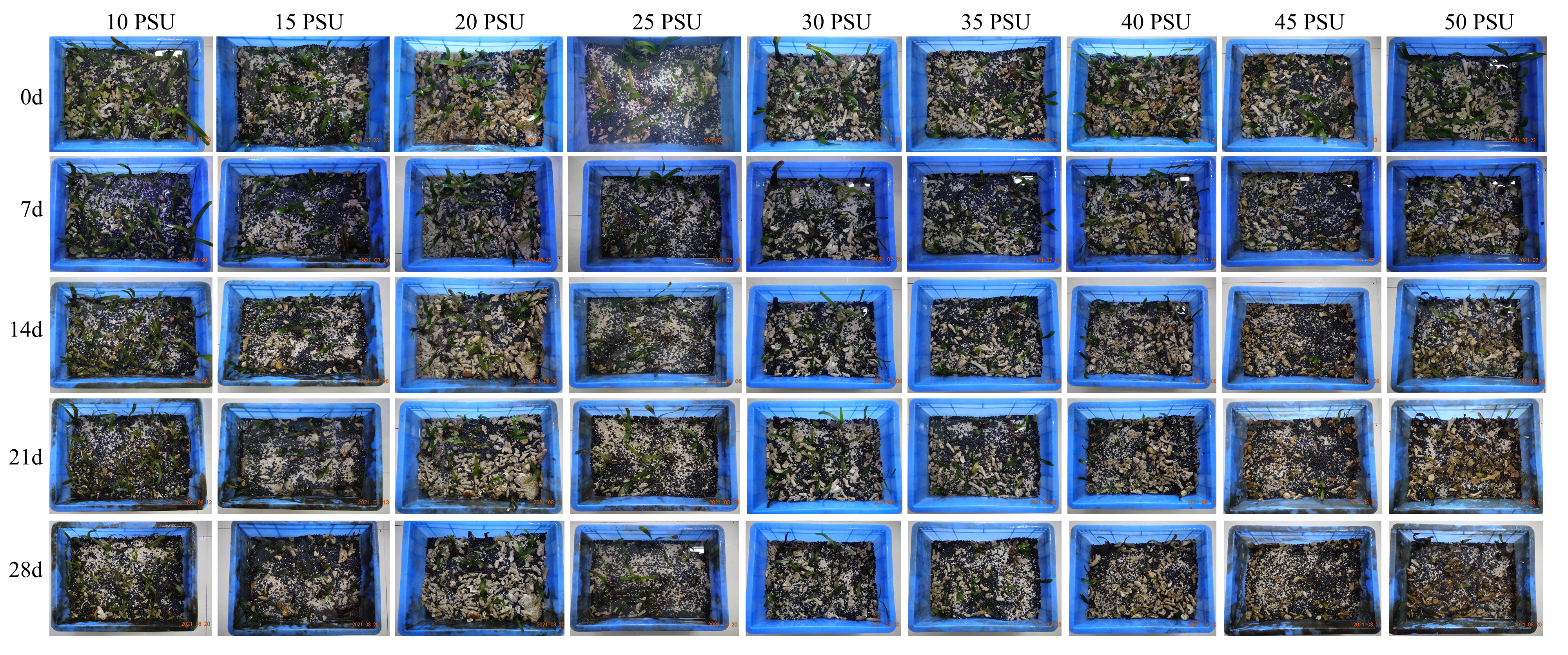

Supplement: Supplementary file 3 [file Image_1.tif]

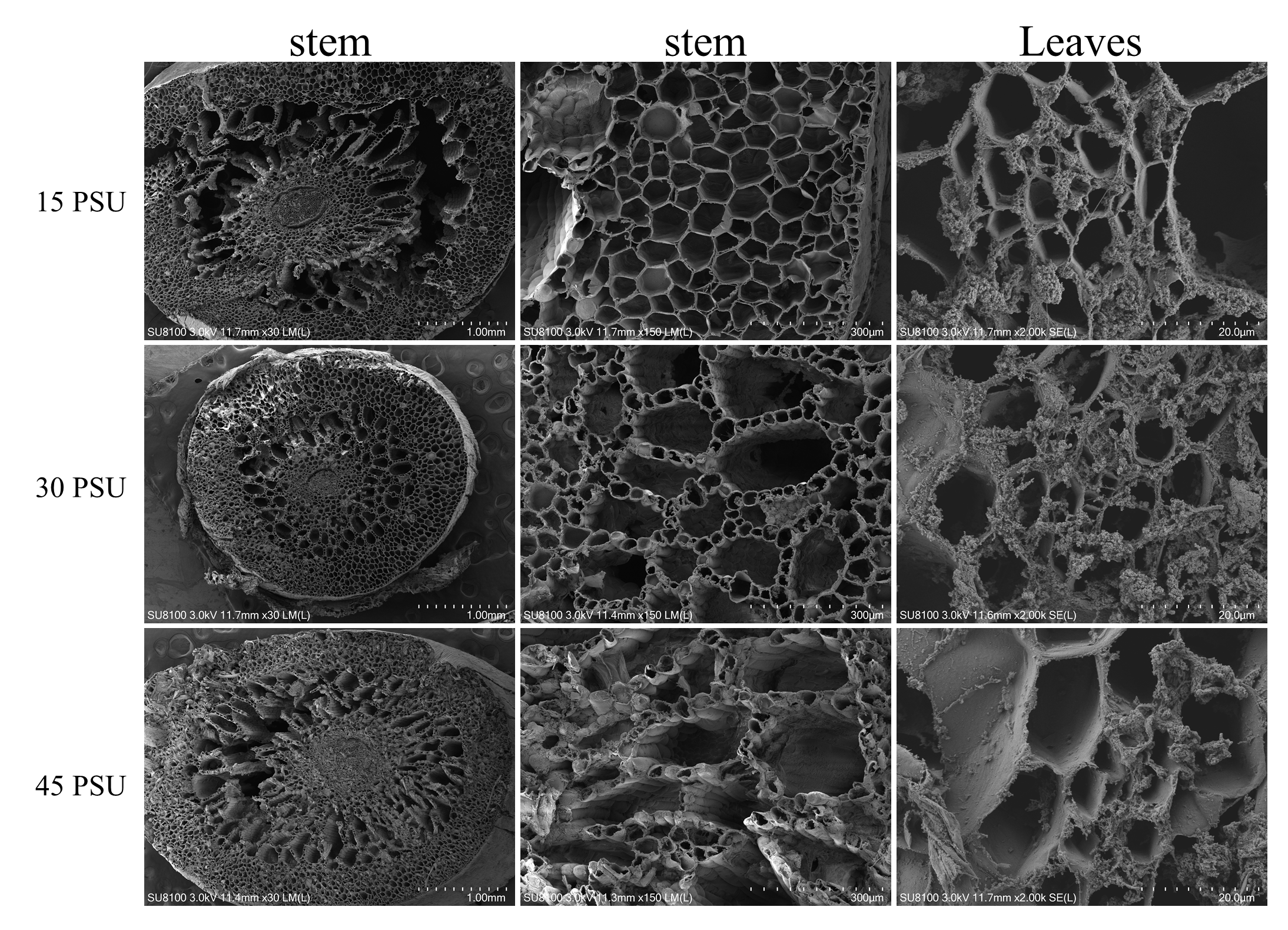

Supplement: Supplementary file 4 [file Image_2.tif]

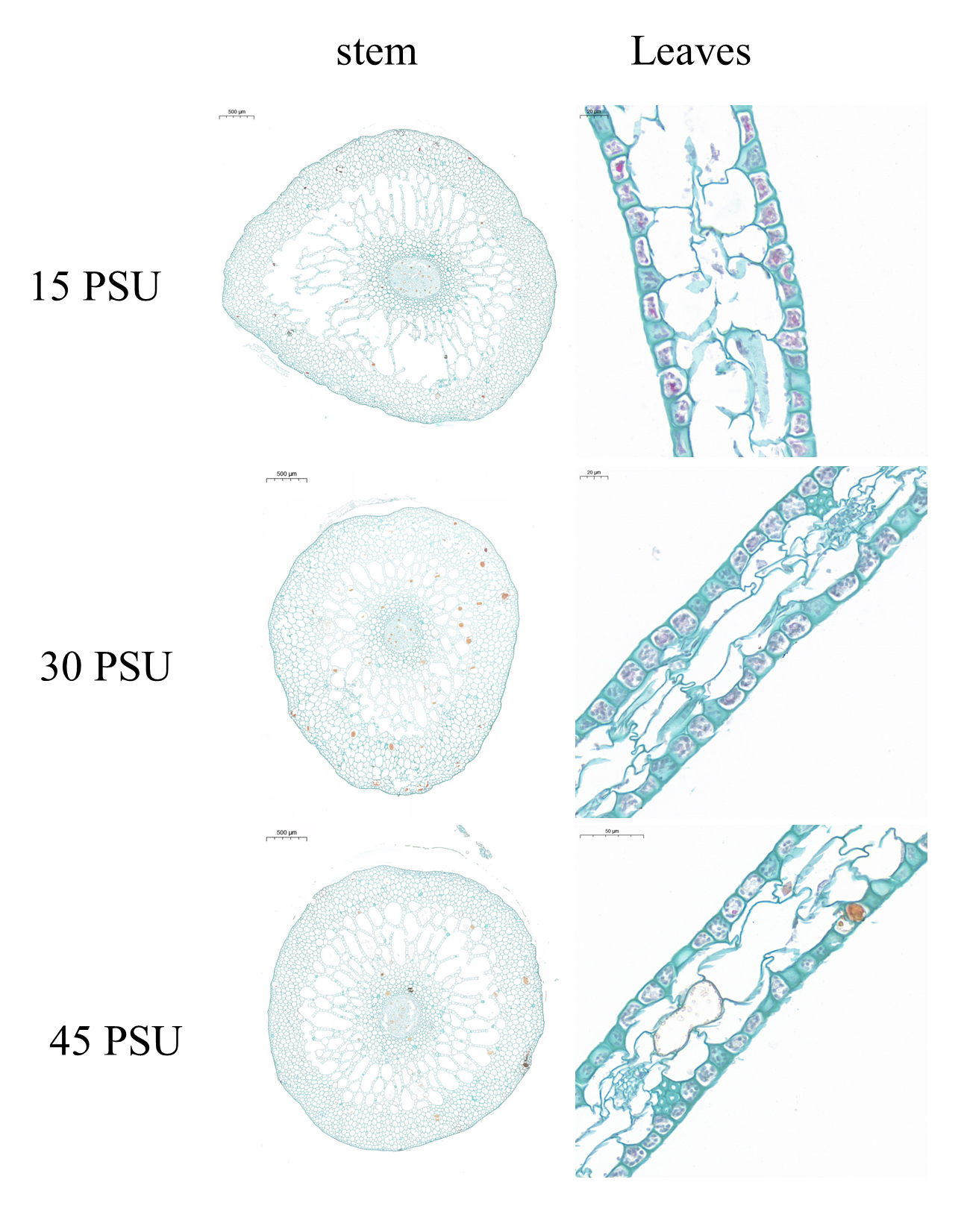

Supplement: Supplementary file 5 [file Image_3.tif]

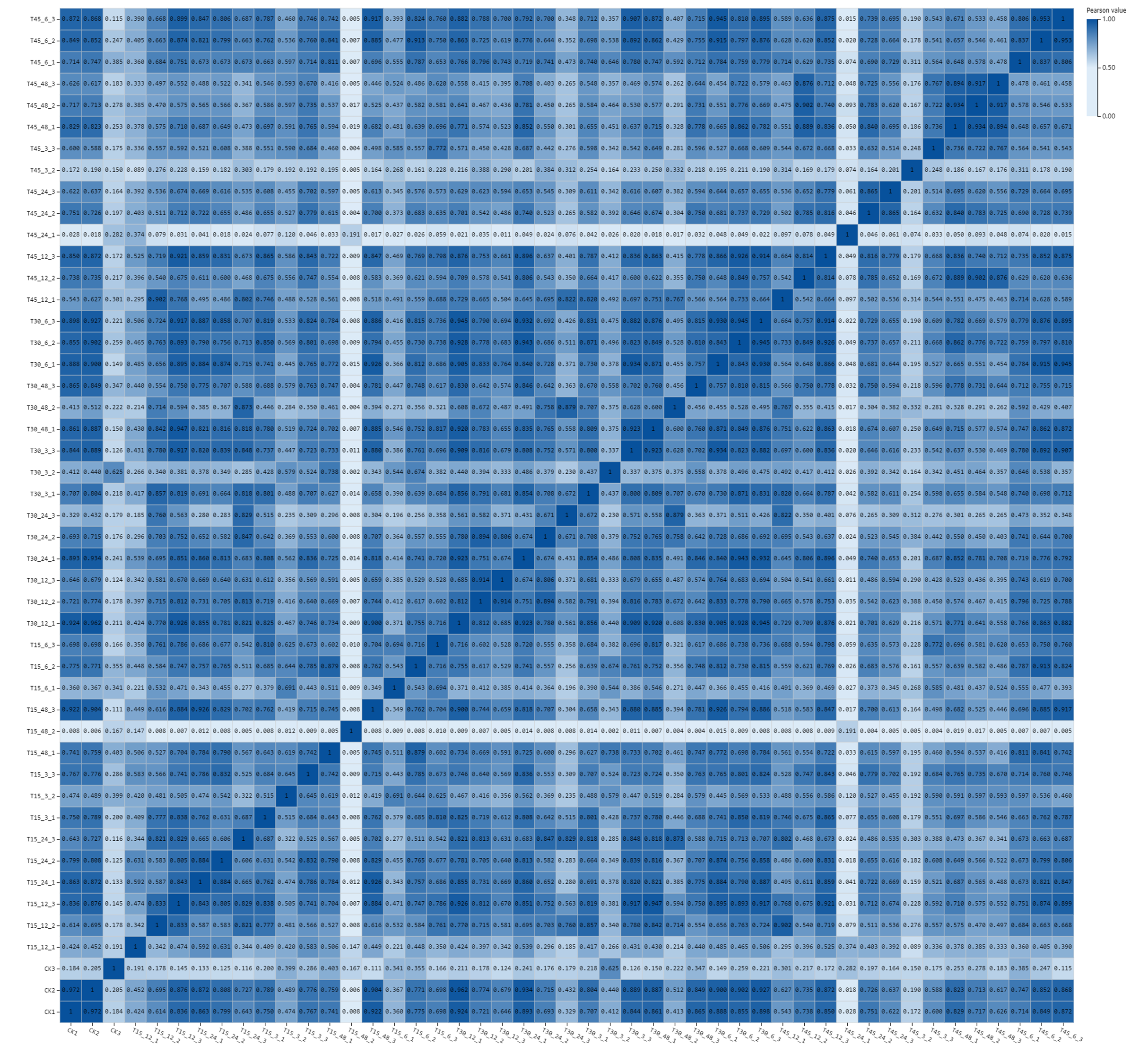

Supplement: Supplementary file 6 [file Image_4.tif]

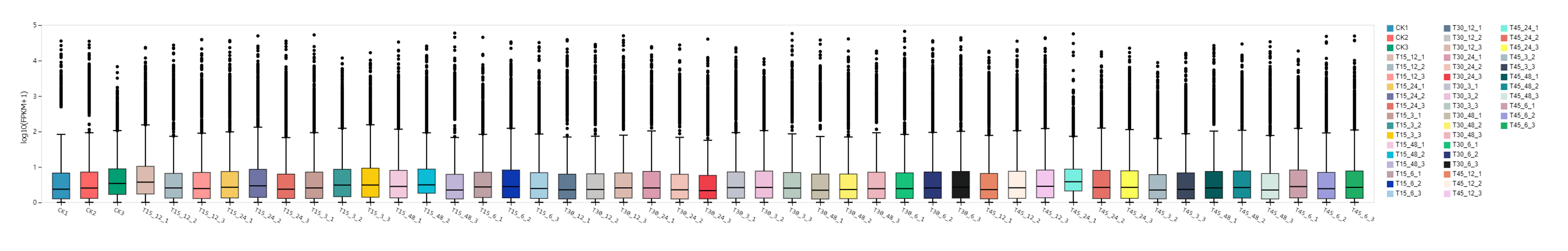

Supplement: Supplementary file 7 [file Image_5.tif]

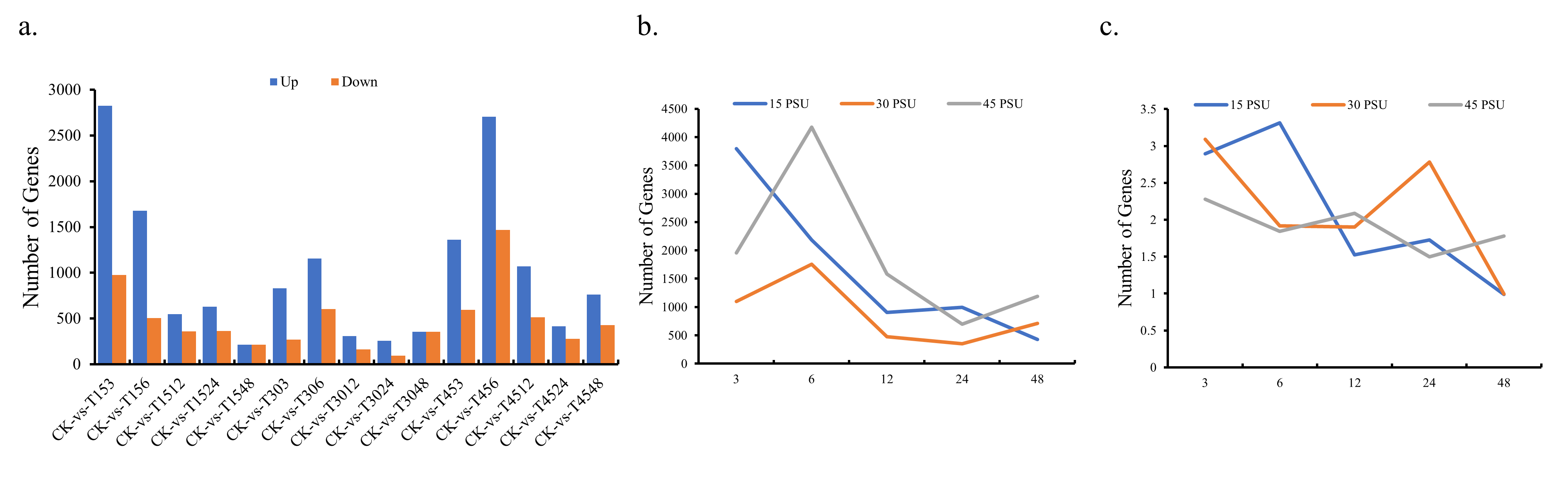

Supplement: Supplementary file 8 [file Image_6.tif]

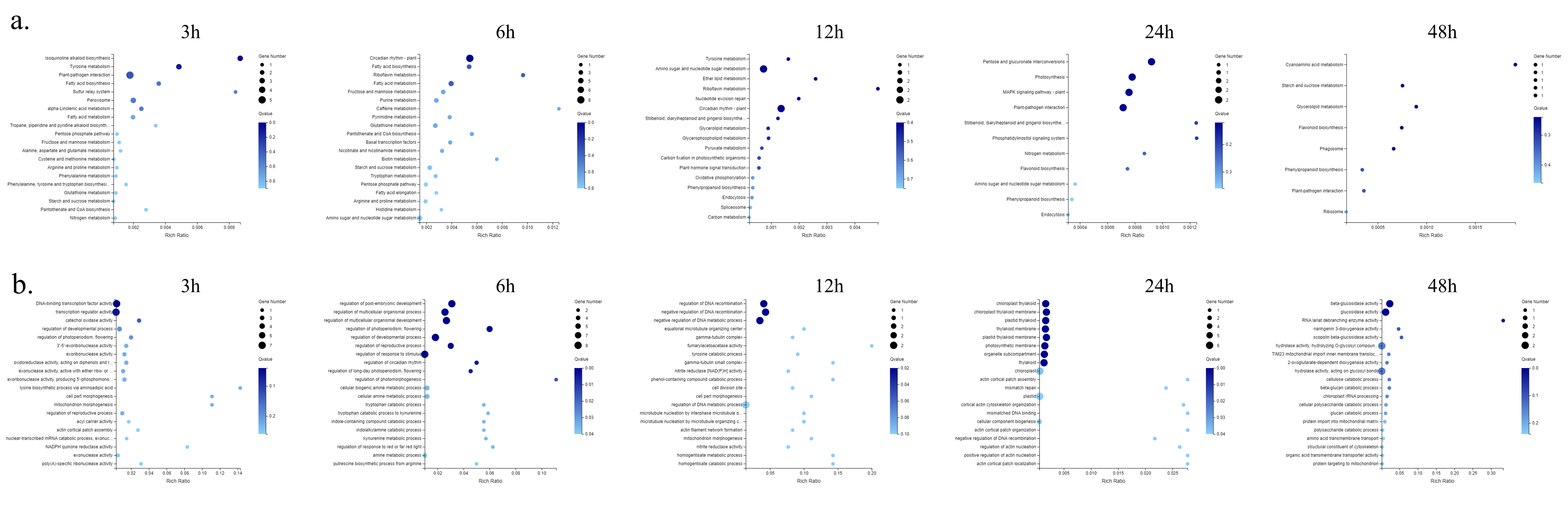

Supplement: Supplementary file 9 [file Image_7.tif]
